# Detection of the Ovarian Cancer Biomarker Lysophosphatidic Acid in Serum

**DOI:** 10.3390/bios10020013

**Published:** 2020-02-14

**Authors:** Brian De La Franier, Michael Thompson

**Affiliations:** Department of Chemistry, University of Toronto, 80 St. George Street, Toronto, ON M5S 3H6, Canada; brian.delafranier@mail.utoronto.ca

**Keywords:** ovarian cancer, lysophosphatidic acid, fluorescence detection, actin, gelsolin

## Abstract

Lysophosphatidic acid (LPA) is present during the medical condition of ovarian cancer at all stages of the disease, and, therefore possesses considerable potential as a biomarker for screening its presence in female patients. Unfortunately, there is currently no *clinically employable* assay for this biomarker. In the present work, we introduce a test based on the duel protein system of actin and gelsolin that could allow the quantitative measurement of LPA in serum samples in a biosensing format. In order to evaluate this possibility, actin protein was dye-modified and complexed with gelsolin protein, followed by surface deposition onto silica nanoparticles. This solid-phase system was exposed to serum samples containing various concentrations of LPA and analyzed by fluorescence microscopy. Measurements conducted for the LPA-containing serum samples were higher after exposure to the developed test than samples without LPA. Early results suggest a limit of detection of 5 μM LPA in serum. The eventual goal is to employ the chemistry described here in a biosensor configuration for the large population-scale, rapid screening of women for the potential occurrence of ovarian cancer.

## 1. Introduction

A patent has been filed on the technology developed in this paper, along with the methods of its production; patent application number PCT/CA2016/050545, and US 15/572,295 by Brian De La Franier and Michael Thompson [1].

In women over 50 years of age, cancers are one of the leading causes of death at over 15% of the population [2,3]. Among female cancers, one of the most dangerous is ovarian cancer. Though ovarian cancer is less common than several other female cancers, such as breast cancer, it displays the highest fatality-to-case ratio of all gynecological cancers, rendering it a very serious issue, especially for post-menopausal women [4,5,6,7,8]. This is reflected in the poor five-year survival rate of 25% for women diagnosed at a late stage of the disease, versus those diagnosed in stages I or II, which is over 90% [9,10,11]. This data clearly implies that there is a critical need to improve diagnosis in the early stages of the disease in order to significantly increase the survival rate for those women who suffer from the disease.

Lysophosphatidic acid (LPA), which is a signaling lipid [12], is a potential ovarian cancer biomarker. In separate studies, this signaling lipid was found to be elevated in 90% of stage I ovarian cancer patients, and 100% of later-stage patients, with concentrations ranging between 1.3 and 50 μM being associated with the disease [13,14]. It has also been linked as a potential biomarker for ovarian cancer, with sensitivity and specificity of over 90%, with a cut-off of 1.3 μM for cancer patients [15,16]. It was also found that LPA elevation correlated to the stage of the disease with stage III and IV patients presenting higher LPA serum concentrations than stage I and II patients [14]. Accordingly, these elevated levels indicate that LPA is a very promising marker for the early-stage detection of ovarian cancer.

Commonly, LPA is detected and quantified in samples using standard analytical methods. This includes the use of capillary electrophoresis and ultraviolet detection [17,18], gas chromatography paired with flame atomic absorption spectroscopy or mass spectrometry [13,19,20,21], thin-layer chromatography paired with mass spectrometry [15,22,23,24,25], liquid chromatography paired with mass spectrometry [14,26,27,28,29,30] or absorbance spectroscopy [31] and matrix-assisted laser desorption/ionization mass spectrometry [32,33,34]. Although these techniques are very sensitive towards LPA, they almost always require lipid extraction or other work-up of the serum samples before use. They also are highly labor intensive, time consuming, and use highly sophisticated instrumentation to quantify the marker. As such, these technologies are inappropriate for practical clinical analysis and, therefore, new methods that use simpler protocols and that are rapid and easy to perform in serum are required for an LPA assay to be included in a large-scale screening protocol for ovarian cancer.

The protein gelsolin [35], which binds to LPA through a small chain of amino acids known as the PIP_2_-binding domain, is eminently capable of acting as a selective probe for the marker [36]. Gelsolin binds LPA with a high affinity measured by its K_d_ of 6 nM [35,37]. The PIP_2_-binding domain, however, only binds LPA with a K_d_ of 920 nM, suggesting that the interactions of LPA and gelsolin are heavily dependent on other components of the protein. The molecule itself is a large six-domain protein, with a molecular mass over 80 kDa [35]. Of the six domains, the protein essentially exists as two identical components comprised of domains 1–3 and domains 4–6, with these halves individually being able to bind to LPA [38]. As such, half of gelsolin, which shall be referred to as gelsolin 1–3, can alone be used as a probe in binding to LPA.

Gelsolin is also an actin-binding protein [39,40,41]. Actin can bind to gelsolin at three different sites and the affinity of this binding depends heavily on which binding site is targeted, and what salts are present. It has been measured with a K_d_ as low as 4.5 × 10^−12^ M, and as high as 400 μM [42,43,44]. Additionally, LPA is a regulator of gelsolin and actin binding, and will cause a release of actin from gelsolin when present in solution [45]. As a result of this release actin can be pre-tagged with a signaling molecule that can be measured in solution following the release of actin.

**Figure 1:**
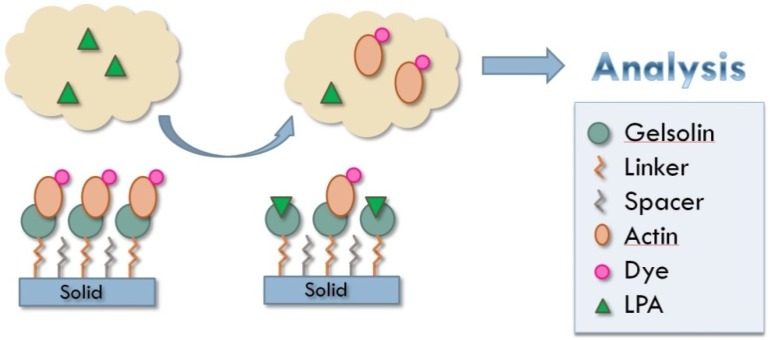
Principle behind actin–gelsolin chemistry for detection of LPA in patient samples. Solid support used in these experiments was silica gel.

In this work, we examined the chemistry of the actin–gelsolin combination for the detection of LPA using fluorescence spectroscopy. By attaching gelsolin to a solid surface, dyed actin can be bound to that surface in a way that is susceptible to release by LPA. Due to the release of actin from gelsolin by LPA, the amount of dyed actin released from the solid into a liquid sample can be directly correlated to the concentration of LPA present. By measuring the amount of dye, and thus the concentration of actin present, the sample concentration of LPA can be determined (Figure 1).

**Figure 2:**
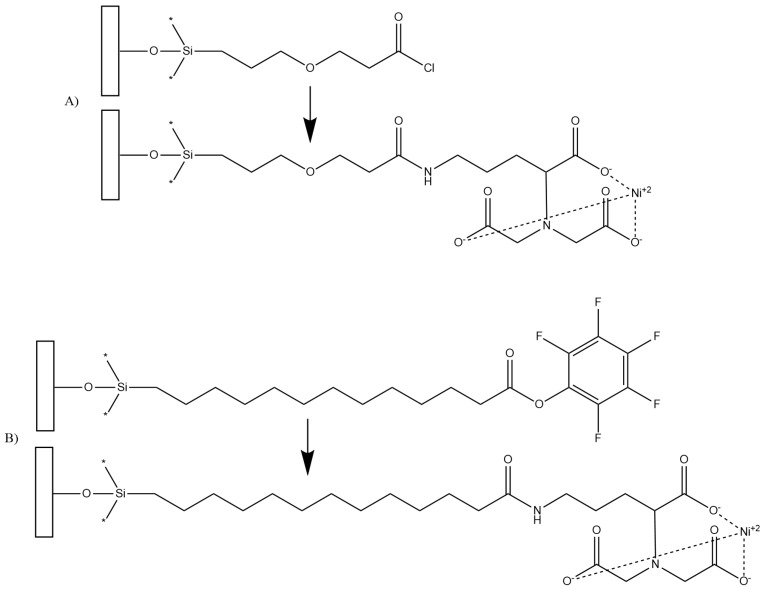
Structures of surface bound 3-(3-(trichlorosilyl)propoxy)propanoyl chloride (MEG-Cl) (**A**) and perfluorophenyl 12-(trichlorosilyl)dodecanoate (PFP-TTTA) (**B**), both before and after extension with Ni-NTA (*N*_α_,*N*_α_-bis(carboxymethyl)-l-lysine with nickel).

In order to develop a test for LPA using these proteins, the molecules must first be bound to a surface. In this work, we employ a trichlorosilane surface linker such as 3-(3-(trichlorosilyl)propoxy)propanoyl chloride (MEG-Cl) [46], or perfluorophenyl 12-(trichlorosilyl)dodecanoate (PFP-TTTA) [47] (Figure 2). These linkers can be bound down to a surface via their trichlorosilane functionality, and extended with *N*_α_,*N*_α_-bis(carboxymethyl)-l-lysine, which can then be employed for binding the polyhistidine-tagged protein form of gelsolin bound to dye modified actin [48]. It is hypothesized that such a test could be developed and will be sensitive to LPA concentration in serum.

## 2. Materials and Methods

### 2.1. Materials

Unless otherwise specified, all materials were purchased from Sigma Aldrich. MEG-Cl and PFP-TTTA were synthesized according to previously published methods [46,47]. Goat serum was used for testing and was purchased from Sigma Aldrich. Gelsolin plasmids were provided by Professor Robert Robinson of the University of Singapore.

### 2.2. Expression of Gelsolin Protein

PSY5 plasmids containing the Gelsolin gene with a histidine tag were introduced to bl21 Rosetta cells for expression. Cells were grown in LB buffer (37.5 g/L LB broth, 10 mg/L ampicillin, 1.5 L). Protein production was induced by isopropyl β-d-1-thiogalactopyranoside (IPTG, 1 mM) during Log phase OD 0.4–0.8 overnight. Cells were pelleted by centrifugation at 4800 rpm for 20 min. Cells were re-suspended in lysis buffer (500 mM NaCl, 20 mM imidazole, 50 mM Tris pH 7.8, 0.1% Triton^TM^ X, 5% glycerol, 1 mg/mL lysozyme, 1 protease inhibitor tablet, 4 mL) and sonicated for 30 min. DNAase I (1 µL) was added to the suspension and the suspension was rocked gently for 30 min. Cell debris was pelleted by centrifugation at 14,500 rpm for 50 min. Gelsolin was purified from the crude solution by use of a Ni-NTA agarose column followed by dialysis into storage solution (20 mM Tris pH 8.0, 0.5 mM EDTA for Gelsolin, 20 mM MES pH 6.0, 1 mM CaCl_2_ for Gelsolin 1–3). Protein mass and purity were determined by SDS-PAGE (12% acrylamide) and concentration by absorbance at 280 nm.

### 2.3. Modification of Actin Protein

Actin from rabbit muscle was purchased from Alfa Aeser or Sigma Aldrich. The Actin (0.3–0.6 mg) was added to acetone (100 μL) and sonicated until broken up into a cloudy precipitate. Buffer A (2 mM Tris-Cl pH 8, 0.2 mM ATP, 0.5 mM 2-mercaptoethanol, 0.2 mM CaCl_2,_ 200 μL) was added to the solution and sonicated for 15 min. To this was added acetone (200 μL) followed by sonication for 5 min. Finally, Buffer A (400 μL) was added to the solution and sonicated until no large solids could be seen. To the solution, now containing 1:2 acetone:Buffer A, NHS-rhodamine in DMF (10 mg/mL, 10 μL) was added. The solution was then shaken under light-free conditions for 90 min, followed by dialysis into buffer B (2 mM Tris-Cl pH 8, 0.5 mM 2-mercaptoethanol, 0.2 mM CaCl_2_, 3.5 mW cutoff). Protein and dye concentrations were determined by absorbance at 280 and 552 nM respectively.

### 2.4. Non-Denaturing Acrylamide Gel Analysis of Proteins

A separating gel (25 mM Tris pH 7.4, 194 mM glycine, 0.2 mM ATP, 0.1% triton-X, 7.5% acrylamide, 0.2% *N*,*N*′-methylenebisacrylamide, 0.03% EDTA, 0.1% ammonium persulfate, 10 mL) was prepared and polymerized with 10% APS (100 μL APS), and TEMED (10 μL) for 20 min. A stacking gel (6% acrylamide, Tris pH 6.8, 5 mL) was prepared, added to the separating gel, and polymerized with 10% APS (100 μL APS), and TEMED (10 μL) for 1 h. The gel was pre-run in electrode buffer (25 mM tris pH 7.4, 194 mM glycine, 0.2 mM ATP, 0.5 mM CaCl_2_) for 30 min at 120 V. Solutions containing gelsolin and actin mixtures were added to the gel with non-denaturing loading dye (225 mM Tris pH 6.8, 45% glycerol, 10 nM bromophenol blue, 1/5 sample volume), and the gel was run at 120 V for 40 min. Proteins were visualized with Coomassie blue. Additionally, the actin–gelsolin complex was exposed to LPA between 1 and 50 μM LPA in PBS buffer for 2 min before being run on another gel using the same method as above.

### 2.5. Cleaning and Surface Modification of Silica Gel

Silica gel, 60 or 150 Å 200–450 mesh, was plasma-oxidized for 1 or 10 min. It was then transferred to a humidity chamber, maintained at 80% relative humidity with a saturated aqueous solution of MgNO_3_·6 H_2_O, and set aside overnight.

Neat MEG-Cl or PFP-TTTA (5 µL) was diluted with anhydrous toluene (5 mL) under inert (N_2_) and anhydrous (P_2_O_5_) atmosphere in a glovebox. A spacer molecule was also added to some versions of the solution, and was either 3-(3-(trichlorosilyl)propoxy)propanoyl chloride (MEG-TFA) or trichloro(hexyl)silane (HTS). The solution was added to glass vials (pre-silanized with trichloro(octadecyl)silane) containing oxidized silica gel (4 mL). The vials were capped and sealed with Parafilm^TM^ M, removed from the glovebox, and placed on a spinning plate for 1.5 h. The silica gel was then rinsed with toluene and sonicated in toluene for 5 min followed by sonication in deionized water for 3 min. The silica was rinsed again with deionized water. A solution of ab-NTA and nickel(II) chloride (2 mg/mL ab-NTA, and 2 mg/mL nickel(II) chloride in deionized water, 4 mL) was added to the silica gel along with pyridine (2 mL) and placed on a spinning plate overnight.

The silica gel, now modified with Ni-NTA, was rinsed with deionized water. Gelsolin and actin solution, which had been pre-incubated for 1 h (0.1 mg/mL each in deionized water, 3 mL) was added to the silica gel and placed on a spinning plate for 1 h. The silica gel was then rinsed with deionized water (50 mL).

### 2.6. Fluorescence Measurements

We performed fluorescence testing in either buffer A solution (pH 8.0) or serum. The following sample solutions were prepared and had their fluorescence measured at the peak absorbance and emission bands for rhodamine dye (λ_ex_ = 552 nm, λ_em_ = 572 nm): solution itself as a negative control, solution with 700 μM imidazole as a positive control, and solutions with between 25 and 100 μM LPA as tests. Then the sample was added to 400 mg of prepared silica gel, incubated for between 0 and 30 min, and filtered. The fluorescence was measured again for each solution, with the difference in fluorescence before and after exposure to the silica gel being the reported signal.

## 3. Results

**Figure 3:**
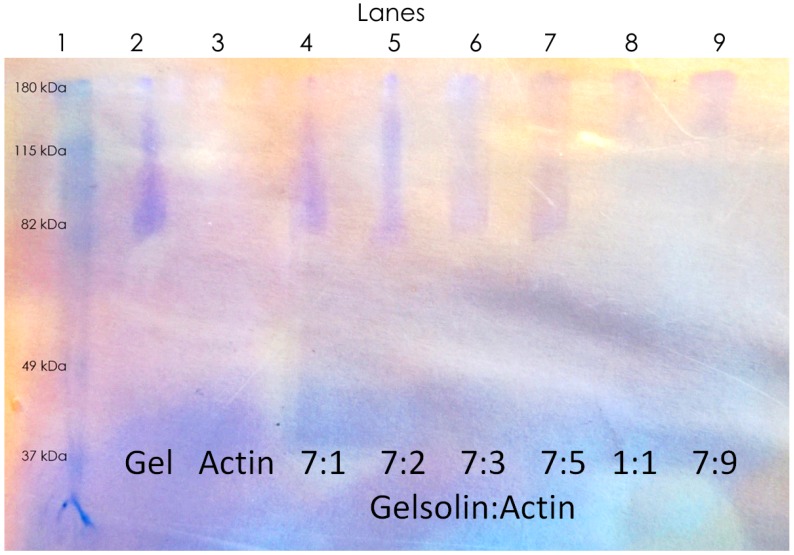
A non-denaturing gel where mixtures of actin and gelsolin were run. Concentration ratios were chosen based on the dissolved concentration of actin.

**Figure 4:**
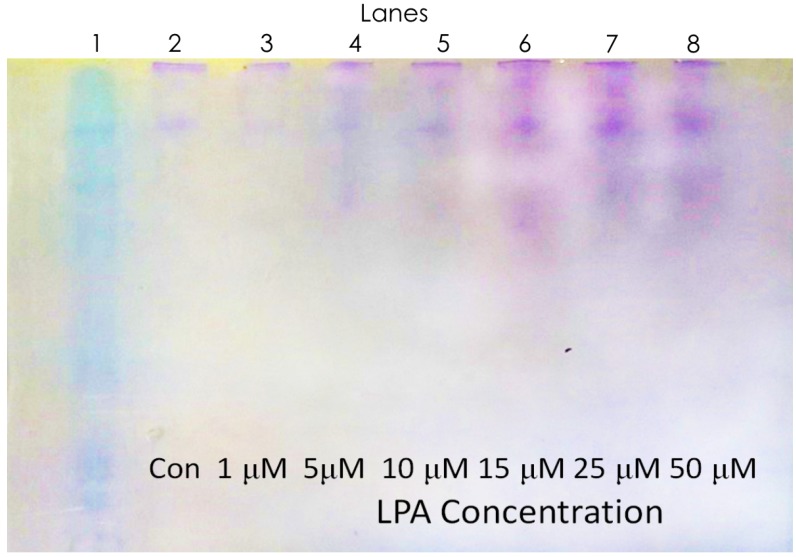
A non-denaturing gel where each lane contains a 1:1 mixture of gelsolin and actin that has been incubated with a varying concentration of lysophosphatidic acid (LPA).

In order for this test to function properly, the modified actin must still be able to bind to gelsolin, and this binding must then be reversed in the presence of LPA. Non-denaturing gels have been used in the past to analyze actin and gelsolin binding [42,44,49]. As such, in order to determine whether dyed actin and the expressed gelsolin are still capable of binding together, these gels were prepared, and two conditions were tested; firstly by making mixtures of modified actin and gelsolin at different ratios in order to determine whether they bind together, and at what ratio this binding was optimal (Figure 3). Secondly by taking the optimal ratio of gelsolin and actin and mixes it with a variety of concentrations of LPA to determine whether LPA is capable of disrupting the actin–gelsolin complex, and whether this disruption is concentration dependent (Figure 4).

**Figure 5:**
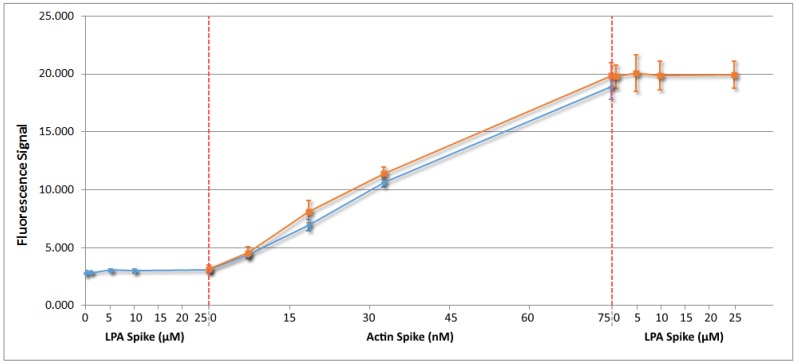
Fluorescence analysis of rhodamine-dyed actin in serum where in orange actin was first added to serum at the specified concentrations followed by the addition of LPA to the final serum solution, and in blue LPA was added to serum at the specified concentrations followed by the addition of actin to the final solution.

In order to determine whether dyed actin is suitable for detection in serum by fluorescence spectroscopy, fluorescence measurements of dyed actin were taken, with and without LPA present in serum (Figure 5). Firstly, rhodamine-dyed actin was introduced to serum (Figure 5, orange trace) and measured. Secondly, LPA was first added to serum followed by the addition of rhodamine-dyed actin (Figure 5, blue trace). This was done to ensure that LPA does not interfere with actin-rhodamine fluorescence.

Several variables in the production of the silica gel testing surface were tested to determine the best way to prepare an actin–gelsolin based test for LPA. These tests were done in buffer A, with variables such as the ratio of surface linker to spacer, the nature of the surface linker, the silica gel pore size, plasma cleaning time, and which version of gelsolin was used being compared. All tests measured the fluorescence signal produced by rhodamine after exposure to the solid surface for 30 s, with the test signal minus the blank being presented (Table 1).

Using the above data, testing was then performed with LPA and imidazole in serum samples. The surface was prepared using the optimal conditions that were found from the above data. This included silica gel with a pore size of 150 Å which was prepared with a 1:2 MEG-Cl:HTS linker system. This was then sonicated in deionized water for 5 min following linker addition. To this was reacted ab-NTA and NiCl_2_ in 2:1 water:pyridine overnight (2 mg/mL). The protein complex of 1:3 gelsolin 1–3:actin-rhodamine was incubated for one hour and added to the now Ni-NTA covered silica gel. After protein addition, the resin was washed with deionized water by vacuum filtration (50 mL) and dried on the Hirsch funnel.

**Figure 6:**
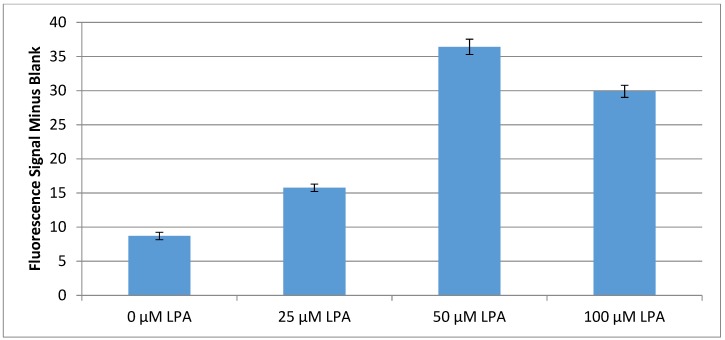
Fluorescence results for silica gel tests incubated in serum samples for 20 min.

Each serum sample tested consisted of 1 mL of sample, to which was added 400 mg of dry modified silica gel, and the resulting solution was mixed by rotation for 20 min, an important improvement to the testing procedure. The solid silica gel was filtered off, and the sample solutions had their fluorescence measured (Figure 6).

## 4. Discussion

### 4.1. Actin and Gelsolin Binding by Gel Analysis

Protein visualization of the non-denaturing gel (Figure 3) presents with a considerable amount of streaking. Unfortunately, due to the limited equipment available to run gels, a small gel run for a relatively short amount of time had to be used. Since there are no denaturing agents present, the proteins move with drastically variable travel times due to packing differences. As a result of this streaking, the actin itself was not visible on the gels. This is most likely due to band widening as compounds progress down the gel. Since actin is much smaller than gelsolin, its band becomes too diluted by this streaking to be visible by Coomassie stain.

Despite the large amount of streaking some conclusions can still be made from the gel. Firstly, gelsolin is visible as a streak that concentrates to 82 kDa as visible in the first lane. The binding of gelsolin and actin is thus visible as the disappearance of this band as the concentration of actin is increased, as well as the appearance of a new band above 120 kDa (the mass of the actin–gelsolin protein complex). It can be seen that at a 1:1 ratio of gelsolin to actin, there is no longer a band visible at 82 kDa (Figure 3, Lane 7), suggesting complete or near complete binding of gelsolin to actin. As such, actin modified with NHS-Rhodamine is still capable of binding to gelsolin, and thus being used as a molecular probe. In order to be useful for the detection of LPA, the dyed actin and gelsolin complex must still be broken apart by LPA, and this must occur in a concentration-dependent manner. Another gel was prepared to investigate this (Figure 4).

As with the previous gel, there is considerable streaking and band widening occurring throughout the gel. However, unlike before, two distinct bands at approximately 120 kDa and greater than 180 kDa are visible in the actin and gelsolin mixture (Figure 4, Lane 2). This suggests that there is a mixture of discrete actin and gelsolin compounds with either 1 or 3 actin molecules bound to the gelsolin.

As LPA is added to the mixture, two new bands begin to appear, one at 82 kDa and one between 120 and 180 kDa. Further, as higher concentrations of LPA are reached, the band between 120 and 180 kDa begins to fade and there is a strengthening of the bands at 82 and 120 kDa. This suggests the loss of actin molecules from the actin–gelsolin compounds, resulting in free gelsolin, and gelsolin bound to 1 or 2 actins. This also strongly suggests that the loss of actin from gelsolin occurs in a concentration-dependent manner and is visible on a gel at a concentration of 5 μM, suggesting that low concentrations of LPA are able to disrupt this complex in an easily detectible manner.

These gels show that not only is gelsolin capable of binding to dye-modified actin, but this complex is sensitive to LPA concentration upon exposure for a very short period of time as the proteins were exposed to LPA for only 2 min prior to being run on the gel. This gives strong evidence for the usefulness of the actin–gelsolin complex in the biosensing of LPA.

### 4.2. Fluorescence Analysis of Dyed Actin and LPA

When rhodamine-dyed actin was introduced to serum, first (Figure 5, orange trace), a linear relationship between the fluorescence signal (λ_ex_ = 552 nm, λ_em_ = 572 nm) and actin concentration was observed. The relationship resulted in a limit of detection of 3.5 nM for the actin concentration. It should be noted that the fluorescence spectrophotometer used in these experiments is an older model which was not optimized. Better fluorescence spectrophotometers would likely sense a much lower concentration of rhodamine-dyed actin.

More importantly, when LPA was added to the serum now containing 75 nM actin, there was no observed change in fluorescence signal at any concentration of LPA up to 25 μM. This suggests that LPA itself will not interfere in a fluorescence test reliant on accurate quantification of rhodamine-dyed actin.

The addition of LPA to the serum did not result in any measurable fluorescence signal up to a concentration of 25 μM (Figure 5, blue trace). The addition of actin into the serum now containing 25 μM LPA was found to cause a linearly increasing fluorescence signal with actin concentration, which was a close match to the relationship found when actin was added to the serum first. As LPA at biologically relevant concentrations has no effect on the ability of actin-rhodamine to fluoresce in serum, dyed actin can be used to test for LPA in serum samples.

### 4.3. Initial Test Development with Buffer A Samples

The initial test development performed in buffer A gave some insight into how this test should be made (Table 1). As can be seen from this data, a ratio of 1:2 linker:diluent ratio produced the highest signal for the imidazole control. Although the signal produced for LPA after subtracting out the negative control of buffer A was slightly lower than the signal observed for a 2:1 ratio, the error was much lower for 1:2 making this ratio more reproducible and thus better for a test. The reason for the 1:2 linker:diluent ratio being superior to pure linker or a 1:1 ratio could be due to protein packing on the surface. Having slightly less linker, and thus Ni-NTA, on the surface could allow for better spacing between proteins preserving their accessibility and function.

As well silica gel with a pore size of 150 Å is preferable to a pore size of 60 Å. This is partly due to a large reduction in non-specific adsorption of the protein complex, evidenced by the much larger signal of 4.2 fluorescence units for the negative control with 60 Å, where under the same conditions a signal of only 0.6 was achieved for 150 Å. Although a larger signal was also seen for the LPA test conditions, this signal is completely swamped by the negative control in the case of silica gel with 60 Å pores. Although the imidazole signal is higher in the case of 60 Å silica gel, this signal is most likely due to non-specifically adsorbed proteins as evidenced by the negative control signals.

The increase in non-specific adsorption for 60 Å silica gel could be in part due to the large size of the full actin–gelsolin complex. The complex has a length of 146 Å across its longest side when one actin is bound [38], making it unable to enter the pores of the 60 Å silica gel. The smaller pores could cause the complex to break apart, allowing the smaller actin, which has a width of 55 Å, to enter the pores and remain on the silica gel in a non-specific manner. As such, the larger pores of 150 Å silica gel would allow the entire complex to enter the pores and bind the protein complex specifically, reducing the negative signal in the absence of LPA.

The results also show that a longer plasma cleaning time is preferable due to not only a higher test signal, but also a reduction in non-specific adsorption of the protein complex, as shown by the higher buffer A signal and lower LPA signal observed for 1 min of plasma cleaning versus 10 mins. This is likely due to insufficient hydroxylation of the surface of the silica gel when plasma cleaned for only 1 min. The lack of hydroxylation would limit the ability of the linker and diluent to bind and form an adlayer, which would also leave patches of bare silica gel exposed. This could increase the amount of non-specific adsorption of the proteins, which causes any signal from LPA to be masked by the negative control. Thus, a plasma cleaning time of at least 10 min should be used in the production of these tests.

Additionally, MEG-Cl as a linker outperformed PFP-TTTA in the signal achieved from buffer A containing LPA by a fairly large margin, with a signal of 1.0 ± 0.3 versus −0.3 ± 0.2, which has a *p*-value of 0.01 (*df* = 3). This could be due to the structure of MEG-Cl, with the interior ether which provides an area for water to bind into the layer [50,51], as well as the more reactive nature of MEG-Cl towards ab-NTA allowing for better coverage of Ni-NTA on the surface of the silica gel. With MEG-Cl as the linker we see a lower signal for the positive imidazole control than we do when PFP is the linker. Although this would suggest that we should see improved performance in the presence of LPA, the opposite is true. The LPA signal observed when MEG-Cl is used as the linker is almost as high as the positive control, suggesting that under the tested conditions that 25μM LPA can saturate the test.

As was discussed previously the first three domains of gelsolin, referred to as gelsolin 1–3, are also capable of binding actin and releasing it in the presence of LPA. As such, another area of interest in improving the surface binding of the protein complex is the difference between gelsolin and gelsolin 1-3 as the anchor protein. The difference in binding and LPA response between gelsolin and gelsolin 1–3 on the surface was thus investigated. This was done using PFP as the linker and HTS as the spacer in a 1 to 2 ratio, as MEG-Cl was unavailable at this time.

As can be seen, both proteins exhibit the same amount of non-specific adsorption as evidenced by the same fluorescence being measured for their negative buffer A controls. However, a larger signal was observed for both LPA and the positive imidazole control when gelsolin 1–3 was the bound protein than when full-length gelsolin was used. This could be due to the smaller size of gelsolin 1–3, allowing it to better pack onto the surface of the silica.

### 4.4. Serum Based Testing and Conclusions

The methods mentioned in the results section for final surface preparation allowed for a strong fluorescence signal to be generated in serum samples containing LPA (Figure 6). A signal difference of 7.2 fluorescence units between the serum sample containing 25 μM and the serum sample with no added LPA was much larger than the previously obtained largest difference of 1.6 fluorescence units. As can be seen, the samples with 50 μM or 100 μM LPA added to them produced even greater signals than this. The 100 μM LPA sample shows a slightly lower fluorescence signal than the 50 μM sample, which suggests a saturation of the test above a 50 μM LPA concentration. This could be due to micelle formation of LPA in the sample as LPA has been shown to have a critical micelle concentration of 50 μM under physiological conditions [52]. Considering the data points of 0, 25, and 50 μM LPA from the above data, and discounting the 100 μM sample due to the clear possibility that micelle formation affected the observed fluorescence, a near linear relationship was found. Using the error in fluorescence of the 0 μM LPA sample, achievement of a limit of detection of 5 μM is evident for the test as currently developed.

## 5. Final Remarks and Future Perspective

The protein complex of actin and gelsolin was found to be highly sensitive to LPA concentration in both buffer and serum samples, leading to a spectroscopy-based biosensing of this key molecule associated with ovarian cancer. The limit of detection indicated in this work at 5 μM LPA in *serum* is close to the value of around 1 μM generally considered to be the basal level in healthy individuals, both male and female. Moreover, the concentrations of LPA in serum examined here are within the range expected for female patients suffering at the various stages of ovarian cancer.

The chemistry described herein leads to attractive future pathways for the detection of ovarian cancer at various stages, including stage 1, both in terms of lowering the limit of detection and possibilities for robotic automation in order to lead to large-scale screening possibilities. With respect to detection, magnetic bead surface chemistry coupled with chemiluminescent measurement offers a feasible direction for the robotic assay of large numbers of samples. Such a development would mandate the transfer of chemistry to Fe surfaces and design of a chemiluminescence method. A further feasible approach is to employ biosensor technology as a transduction mechanism rather than conventional spectroscopy. In this regard, an intriguing strategy would be to use acoustic wave biosensor detection of the nanoparticle-associated chemistry described in the present work in analogous fashion to that employed for gold nanoparticles [53]. Research towards these goals is underway in our laboratory. In conclusion, these efforts are motivated by the 2016 recommendation of the National Academies of Sciences Committee on the State of the Science in Ovarian Cancer Research [54]:

“Researchers and funding organizations should focus on the development and assessment of early detection strategies that extend beyond current imaging modalities and biomarkers and that reflect the pathobiology of each ovarian cancer subgroup”.

## Figures and Tables

**Table 1:** Fluorescence data for initial test development with different variable for surface preparation. Bolded rows were the preferred conditions as given by the overall fluorescence signal for 25 μM LPA in Buffer A.

Variable	Condition	25 μM LPA Signal	Buffer A	Overall	Imidazole
			Signal	Signal	Signal
**Linker: Spacer Ratio**	1PFP:1HTS	0.27 ± 0.07	0.23 ± 0.06	0.04 ± 0.07	1.4 ± 0.2
	2PFP:0HTS	0.32 ± 0.06	0.17 ± 0.06	0.15 ± 0.06	0.8 ± 0.2
	**2PFP:1HTS**	**0.9 ± 0.3**	**0.5 ± 0.2**	**0.4 ± 0.3**	**3.0 ± 0.3**
	**1PFP:2HTS**	**0.61 ± 0.04**	**0.40 ± 0.05**	**0.21 ± 0.05**	**4.3 ± 0.4**
**Silica Pore Size**	60Å	3.0 ± 0.5	4.2 ± 0.5	−1.2 ± 0.5	9.8 ± 0.6
	**150Å**	**0.9 ± 0.2**	**0.6 ± 0.2**	**0.3 ± 0.2**	**4.3 ± 0.4**
**Plasma cleaning time**	1 min	0.7 ± 0.2	1.1 ± 0.2	−0.4 ± 0.5	4.1±0.4
	**10 min**	**1.0 ± 0.2**	**0.7 ± 0.2**	**0.3 ± 0.2**	**3.2 ± 0.2**
**Linker**	PFP-TTTA	0.0 ± 0.2	0.3 ± 0.2	−0.3 ± 0.2	3.2 ± 0.2
	**MEG-Cl**	**2.2 ± 0.3**	**1.2 ± 0.2**	**1.0 ± 0.3**	**2.3 ± 0.5**
**Protein**	Gelsolin	0.2 ± 0.2	0.6 ± 0.3	−0.5 ± 0.3	1.3 ± 0.3
	**Gelsolin 1–3**	**0.9 ± 0.1**	**0.6 ± 0.2**	**0.3 ± 0.2**	**2.7 ± 0.2**
